# Is Neutrophil-to-Lymphocyte Ratio a Predictor of Coronary Artery Disease in Western Indians?

**DOI:** 10.1155/2017/4136126

**Published:** 2017-07-24

**Authors:** Kamal Sharma, Alap K. Patel, Komal H. Shah, Ashwati Konat

**Affiliations:** ^1^Department of Cardiology, U. N. Mehta Institute of Cardiology and Research Centre, Ahmedabad, Gujarat, India; ^2^Department of Research, U. N. Mehta Institute of Cardiology and Research Centre, Ahmedabad, Gujarat, India

## Abstract

**Introduction:**

The current study was designed to evaluate the association of neutrophil-to-lymphocyte ratio (NLR) with coronary artery disease (CAD) presence. We also aimed to propose a suitable cut-off of NLR for diagnosis of CAD in Western Indians.

**Methods:**

Total 324 patients undergoing coronary angiography were enrolled and were subdivided into two groups: group 1 (*n* = 99; population without CAD) and group 2 (*n* = 225; population with CAD).

**Results:**

The results indicated significant (*p* < 0.05) positive association between elevated levels of WBC, neutrophil, monocyte, NLR, hs-CRP, CPK-MB, and troponin I and disease presence. According to subgroup analysis, the association was more profound in male and older population. Among all the markers NLR showed the strongest predictive potential for CAD with highest odds ratio (1.495; 95% CI: 0.942–2.371; *p* < 0.048). Optimum cut-off of NLR for diagnosis of CAD was 2.13 (AUC-0.823; *p* < 0.001; sensitivity: 83.64%; specificity: 63.46%). Association of NLR with other biochemical markers such as hs-CRP, CPK-MB, and troponin I was also observed in quartile analysis.

**Conclusion:**

NLR is a simple indicator that could be effectively used for the diagnosis of CAD with a cut-off of 2.13 in Western Indian population.

## 1. Introduction

The relationship between various inflammatory markers and coronary artery disease (CAD) has been established long ago [1]. Among them, white blood cell (WBC) subtypes have immerged as a community of inflammatory markers playing a crucial role in the pathogenesis of atherogenesis and atherothrombosis [2]. Neutrophil-to-lymphocyte ratio (NLR)—a new addition to the long list of markers—is an inexpensive, easy to obtain, widely available marker of inflammation, which can aid in the risk stratification of patients with various cardiovascular diseases in addition to the traditionally used markers. Ample research databases from Indian subcontinents have supported a potential of NLR as a prognostic and diagnostic index of coronary artery disease (CAD) and disease associated mortality [3–5]. An elevated NLR, irrespective of other biomarker levels, independently indicates an increased long term risk of mortality not only in patients with stable CAD but also in acute coronary syndrome (ACS) patients [6, 7].

These studies substantiate the negative impact of elevated NLR, and hence effort had been made to propose a suitable cut-off of it with effective clinical usage in various patient population. However the reference values for NLR vary with age and ethnicity. Misumida et al. in 2015 had also demonstrated an independent association between race and NLR in patients with NSTEMI, suggesting that a tailored cut-off value according to race would provide more precise prognostic information. These variations need to be considered while using NLR for predictive and prognostic purposes and before proposing a diagnostic cut-off in particular race. While some studies categorized their patients according to NLR intervals (e.g., tertiles, quartiles, and quintiles) [8–10], other studies used definite NLR cut-off points (e.g., NLR ≥ 2.5, NLR ≥ 2.7, NLR ≥ 3, NLR ≥ 4), and others used NLR ≥ 5 [11–16]. In fact, studies report different timing for the collection of blood used to calculate NLR; some collect the blood sample on admission [7], and others use preoperative NLR [17], maximum NLR during hospitalization [18], or average NLR of three readings during hospitalization [19].

Herewith we aim to investigate the association of NLR with CAD and establish it as a useful diagnostic and prognostic tool for CAD in Western Indians. We also seek to propose a suitable cut-off of NLR with effective clinical usage in this population.

## 2. Materials and Method

### 2.1. Study Population

This prospective study was conducted at U. N. Mehta Institute of Cardiology and Research Centre and was approved by institutional ethics committee. Total 324 individuals of both genders were enrolled from March 2014 to January 2016. The patients admitted for coronary angiography (CAG), hospitalized with first-time chest pain, having myocardial infarction, and with ECG showing changes and patients admitted in emergency were included in this study. Exclusion criteria of the study were as follows: patients taking any lipid lowering drug (statin), recent major surgery, and rheumatic heart disease. All patients were evaluated by taking detailed history and physical examination. The variables included in the study were age, sex, hypertension (HTN), diabetes mellitus (DM), smoking, cardiac biomarkers (troponin I, CK-MB), hs-CRP and white blood cell (WBC) count, differential count, mean platelet volume (MPV), red cell distribution width (RDW), and erythrocyte sedimentation rate (ESR). Hypertension was defined as the active use of antihypertensive drugs or documentation of blood pressure more than 140/90 mmHg, and diabetes mellitus was defined as fasting plasma glucose (FPG) levels over 126 mg/dl or random plasma glucose level over 200 mg/dl or active use of antidiabetic treatment. Smoking was defined as current smoking status of an individual. Complete blood count and biochemical values were evaluated from blood samples obtained by antecubital vein puncture. The study population was divided into two groups based on angiographic findings: group 1 (*n* = 99; population without CAD – no/nonsignificant CAD) and group 2 (*n* = 225; population with CAD - stenosis >70%).

### 2.2. Biochemical Estimations

Blood samples for laboratory assessment were collected upon first point of patient contact to refrain from bias. Total leucocyte count and its subtypes including neutrophil and lymphocyte platelet count and MPV and RDW were analysed using an automated blood cell counter, CELLDYN Ruby (Abott). Troponin I, CPK-MB, and hs-CRP were analysed using counter ARCHITECT PLUS (ci 4100) (Abott). Erythrocytes sedimentation rate was assessed using E-VACC disposable ESR pipettes (matrix) at 1 hr. Troponin I, CPK-MB, hs-CRP, CBC, and ESR were assessed from the same sample.

### 2.3. Statistical Analysis

All statistical studies were carried out using SPSS program version 20. Neutrophil-to-lymphocyte ratio (NLR), WBC-to-platelet ratio (WBCPR), and platelet-to-lymphocyte ratio (PLR) were automatically calculated by loading all the data to the statistical program used. Quantitative variables were expressed as the mean ± standard deviation and qualitative variables were expressed as percentage (%). Univariate statistics between two groups were calculated using the *t*-test or Mann–Whitney *U* test whichever is applicable. Categorical variables were compared using the chi-square test. A correlation between the variables was determined by using Pearson's correlation test. Strength of an association of the markers with disease presence was assessed using logistic regression for the parameters showing association with disease presence on univariate analysis (*p* < 0.05). Receiver operating characteristics (ROC) curves were constructed and the most discriminating cut-off values were determined to assess the predictive value of the NLR. Distribution of NLR values in the quartile ranges of established cardiac biomarkers were also calculated. A level of significance was accepted as a two-tailed *p* value < 0.05.

## 3. Results

The comparative baseline details of all the biochemical parameters between the two groups are presented in Table 1. Elevated levels of WBC count and neutrophil and monocyte count were observed in group 2 patients as compared to group 1 population. Significantly (*p* < 0.05) low level of NLR was found in group 1 (4.3 ± 3.8) in contrast to group 2 (5.6 ± 4.5). Patients with significant CAD had higher RDW (12.9 ± 1.6) as compared to patients without CAD (12.4 ± 2). Raised hs-CRP (3.3 ± 4.3 versus 1.8 ± 4.2), CPK-MB (116.4 ± 152.5 versus 51.4 ± 76.7), and troponin I (14 ± 18.8 versus 6.3 ± 14.4) levels were also found in group 2 patients as compared to group 1 subjects. The relationship between CAD and biochemical markers according to age and gender are presented in Tables 2 and 3. NLR showed a significant association with CAD in male and comparatively older (>40 years) population. Nonsignificantly (*p* = 0.333) high mean NLR value was found in patients with ejection fraction (EF) of <50% (5.55 ± 5.04) as compared to patients with EF of ≥50% (4.75 ± 3.19) in CAD positive group.

**Figure 1:**
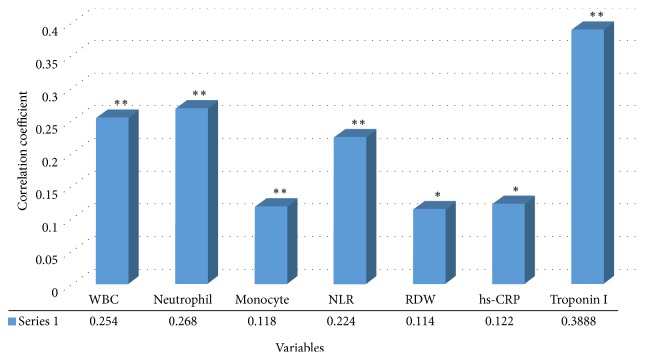
Correlation of biochemical markers with disease presence. ^*∗∗*^Correlation is significant at the 0.01 level (2-tailed). ^*∗*^Correlation is significant at the 0.05 level (2-tailed). WBC: white blood cell, NLR: neutrophil-to-lymphocyte ratio, RDW: red cell distribution width, and hs-CRP: high sensitivity C-reactive protein.

Figure 1 shows correlation analysis of various markers with disease presence. Strong positive correlation was observed between increasing values of WBC, neutrophil, monocyte, NLR, and troponin I and CAD occurrence. The strength of association of the markers with CAD was assessed using regression analysis and is presented in Table 4. Among all the studied variables NLR was found to be the strongest predictor of CAD showing an odds ratio of 1.495 (95% CI: 0.942–2.371; *p* = 0.048). Following this the diagnostic potency of the significantly associated markers were investigated using ROC analysis (Table 5). As indicated NLR exhibited highest area under curve (AUC-0.823; *p* = 0.0001; 95% CI; 0.712–0.931), closely followed by neutrophil count (AUC-0.821; *p* = 0.0001; 95% CI; 0.714–0.932) and troponin I (AUC-0.820; *p* = 0.0001; 95% CI; 0.716–0.935). Based on ROC a suitable cut-off of NLR was found to be 2.13 showing sensitivity and specificity of 83.64% and 63.46%, respectively (Table 6). According to quartile analysis, as indicated in Table 7 with increasing quartile of ACS markers there is an increase in NLR mean value. The association between NLR and left ventricle ejection fraction (LVEF) was assessed using Pearson's correlation analysis in CAD patients and results showed that there is no significant correlation between both parameters (coefficient of correlation: −0.086; *p* = 0.234).

## 4. Discussion

To our knowledge, this study is the first to propose clinically most relevant cut-off of NLR with considerably high sensitivity and specificity of CAD in Western Indians. We herewith demonstrate that patients with abnormal CAG had significantly higher NLR compared to patients with normal CAG.

The NLR test, which can be derived from the WBC count, is a common, cheap, and reproducible test worldwide. Previous studies have shown that NLR is associated with poor clinical outcomes in various cardiovascular diseases [8, 20–22]. Through entire spectrum of CAD and its associated diseases, the role of NLR has been extensively studied in order to provide a cheap and easily accessible for screening of population at risk. Following global trend, from India too various studies have proposed NLR cut-off for diagnosis of CAD. Fernando et al. (2015) had studied the relation of NLR with CAD in diabetic population and found ≥ 2.26 as the best suitable cut-off to identify the presence of CAD in diabetic patients [4]. Among immune-inflammatory markers in non-ST-elevation acute coronary syndrome and stable angina patients also, neutrophil counts and NLR were significantly correlated with noncalcified plaques, suggesting the potency of these easily measured biomarkers in reflecting the burden of vulnerable plaques in CAD. Numerous imaging modalities such as invasive coronary angiography and calcium scoring by multidetector CT have also confirmed the role of NLR in the presence, severity, and progression of coronary atherosclerosis [23, 24]. Parallel to us Sari et al. (2015) have also reported that from all other systemic inflammatory markers only NLR is the predictor of CAD showing a strong odds ratio (1.576, confidence interval: 1.198–2.072, *p* = 0.001) [25]. Even in geriatric population too, the patients with CAD had higher NLR, where the cut-off of 1.96 was reported with 66.5% of sensitivity and 48.8% of specificity (AUC = 0.575) [26]. In addition, higher NLR has also been associated with increased cardiac mortality in clinically stable patients with CAD compared with total WBCs count [27]. In patients with chronic coronary total occlusion (CTO), the NLR was significantly higher showing a positive correlation with SYNTAX score. The cut-off identify for CTO disease by NLR was 2.09 with a sensitivity and specificity of 61% and 69.3%, respectively. Similarly in this study NLR was found to be the strongest predictor of CAD with highest odds ratio; however we are able to achieve greater degree of sensitivity and specificity as compared to previous reported studies. The improvement in sensitivity could be obtained by lowering the cut-off; however in that case we were compromising on specificity and hence the current cut-off of 2.13 was found to be most suitable for Western Indians.

The main role of neutrophilia in CAD may be explained by secretion of various inflammatory mediators such as elastase, myeloperoxidase, and oxygen free radicals which causes tissue damage. The probable cause of lymphopenia include decreased production as a result of increased steroid level due to CAD induced stress and increased apoptosis triggered by increased inflammation thereby resulting in elevated NLR in CAD (+) group [2, 28]. Increased number of neutrophils and decreased lymphocytes are risk indicators for future cardiovascular events. Therefore, elevated NLR integrates the predictive risk of the two leukocyte subtypes into a single risk factor. As the biomarkers used in this study predicted cardiac myocyte damage, the possibility of their association with LVEF was also evaluated in order to differentiate between heart failure patients with preserved EF and heart failure patients with depressed EF. In concordance with other studies where higher NLR values were associated with lower EF [29], in our population also patients with low EF had elevated mean NLR value as compared to their counterparts, though in our case the difference could not reach a statistically significant level. Moreover the lack of association was also observed between overall EF and that of NLR values. This finding could be justified by the fact that greater number of the patients (≈60%) had compromised LVEF and hence more systematic randomized study is needed to evaluate association of LVEF with NLR in Indian CAD patients.

Diagnosis of CAD is principally based on CAG and other cardiovascular imaging modalities; however these tools are expensive and time consuming with potential unwanted effects such as exposure to radiation. Therefore, NLR, which is cheap and easily obtainable, could be used as an initial filter criterion, especially in small centres to determine the need for further imaging modalities in the assessment of CAD.

## 5. Conclusion

Keeping in mind the early acting immunological nature of neutrophils and lymphocytes and their presence in the blood circulation, we recommend the use of NLR as a biomarker of CAD because of their simple, easily measurable, and inexpensive method. Along with previous international recommendations, our study supports the use of NLR as a cost-effective biomarker to predict the future cardiovascular risk.

## Figures and Tables

**Table 1:** Comparison of parameters between group 1 (population without CAD) and group 2 (population with CAD).

Variables	Groups	Mean	Standard deviation	Significance
WBC	Group 1	10175.6	3772.0	0.034
	Group 2	12045.8	4596.6	
Neutrophil	Group 1	7284.2	3924.9	0.041
	Group 2	9184.2	4532.9	
Lymphocyte	Group 1	2265.6	951.9	0.507
	Group 2	2191.0	922.6	
Eosinophil	Group 1	257.4	169.3	0.391
	Group 2	254.8	190.2	
Monocyte	Group 1	370.1	304.5	0.0001
	Group 2	476.9	209.1	
Basophil	Group 1	0.7	6.5	0.404
	Group 2	1.3	13.9	
Platelet	Group 1	308440.0	120954.6	0.818
	Group 2	318787.4	159314.1	
MPV	Group 1	6.6	1.3	0.150
	Group 2	6.9	1.5	
NLR	Group 1	4.3	3.8	0.001
	Group 2	5.6	4.5	
PLR	Group 1	163.4	107.9	0.751
	Group 2	190.8	266.4	
RDW	Group 1	12.4	2.0	0.002
	Group 2	12.9	1.6	
hs-CRP	Group 1	1.8	4.2	<0.0001
	Group 2	3.3	4.3	
CPK-MB	Group 1	51.4	76.7	<0.0001
	Group 2	116.4	152.5	
Troponin I	Group 1	6.3	14.4	<0.0001
	Group 2	14.0	18.8	
ESR	Group 1	22.4	8.3	0.227
	Group 2	23.6	8.5	

CAD: coronary artery disease, Group 1: population without CAD, group 2; population with CAD, WBC: white blood cell, MPV: mean platelet volume, NLR: neutrophil-to-lymphocyte ratio, PLR: platelet-to-lymphocyte ratio, PWBC: platelet white blood cell ratio, RDW: red cell distribution width, hs-CRP: high sensitivity C-reactive protein, CPK-MB: creatinine phosphokinase-MB, and ESR: erythrocyte sedimentation rate.

**Table 2:** Comparison of parameters between group 1 (population without CAD) and group 2 (population with CAD) according to gender.

Variables	Groups	Male			Females		
		Mean	Standard deviation	Significance	Mean	Standard deviation	Significance
WBC	Group 1	10214.9	3783.8	0.002	10065.1	3810.9	0.032
	Group 2	12014.7	4598.9		12338.9	4479.7	
Neutrophil	Group 1	7337.3	3915.8	0.002	7135.2	4024.3	0.039
	Group 2	9168.0	4550.5		9293.0	4446.1	
Lymphocyte	Group 1	2241.2	972.0	0.667	2334.0	908.0	0.978
	Group 2	2184.7	928.7		2328.6	737.5	
Eosinophil	Group 1	285.2	164.1	0.272	266.6	141.0	0.838
	Group 2	277.8	184.1		276.0	180.3	
Monocyte	Group 1	380.6	171.9	0.477	435.8	525.5	0.008
	Group 2	409.1	214.1		443.9	175.7	
Basophil	Group 1	1.9	7.6	0.658	0.7	6.5	0.65
	Group 2	2.6	14.2		3.6	16.3	
Platelet	Group 1	293062.5	136846.6	0.979	335777.8	86389.8	0.953
	Group 2	276300.0	81127.3		320840.0	62082.4	
MPV	Group 1	6.7	1.4	0.382	6.4	1.0	0.145
	Group 2	6.9	1.5		7.0	1.5	
NLR	Group 1	4.5	4.0	0.011	3.9	3.4	0.055
	Group 2	5.2	3.7		4.5	2.8	
PLR	Group 1	153.1	104.8	0.431	181.8	117.2	0.626
	Group 2	157.2	79.3		146.7	52.3	
RDW	Group 1	12.7	2.1	0.89	11.7	1.4	0.006
	Group 2	12.5	1.5		12.4	1.6	
hs-CRP	Group 1	1.6	3.9	0.382	2.5	5.1	0.284
	Group 2	1.8	3.3		3.6	6.6	
CPK-MB	Group 1	48.9	57.4	<0.0001	58.5	116.2	<0.0001
	Group 2	111.6	141.8		106.9	136.4	
Troponin I	Group 1	6.0	13.8	<0.0001	7.0	16.3	0.001
	Group 2	13.4	18.7		15.8	19.0	
ESR	Group 1	22.5	9.1	0.243	22.1	6.0	0.803
	Group 2	23.6	8.4		23.5	9.2	

CAD: coronary artery disease, group 1: population without CAD, group 2; population with CAD, WBC: white blood cell, MPV: mean platelet volume, NLR: neutrophil lymphocyte ratio, PLR: platelet lymphocyte ratio, PWBC: platelet white blood cell ratio, RDW: red cell distribution width, hs-CRP: high sensitivity C-reactive protein, CPK-MB: creatinine phosphokinase-MB, and ESR: erythrocyte sedimentation rate.

**Table 3:** Comparison of parameters between group 1 (population without CAD) and group 2 (population with CAD) according to age.

Variables	Groups	≤40 years			>40 years		
		Mean	Standard deviation	Significance	Mean	Standard deviation	Significance
WBC	Group 1	8795.7	2103.3	0.17	10280.6	3856.5	<0.0001
	Group 2	10804.9	3617.9		12267.8	4665.6	
Neutrophil	Group 1	5921.5	2256.7	0.168	7387.9	4012.5	<0.0001
	Group 2	7896.2	3511.4		9381.9	4623.7	
Lymphocyte	Group 1	2378.6	1018.8	0.889	2257.0	952.0	0.619
	Group 2	2316.5	1055.6		2200.6	868.1	
Eosinophil	Group 1	202.8	84.8	0.282	286.5	160.7	0.195
	Group 2	304.6	239.0		273.8	174.6	
Monocyte	Group 1	292.8	62.8	0.454	402.9	314.3	0.058
	Group 2	343.1	171.3		427.2	209.5	
Basophil	Group 1	0	0		1.2	12.7	0.653
	Group 2	9.8	37.6		3.9	56.9	
Platelet	Group 1	358000.0	141421.4	0.532	304130.4	121689.0	0.972
	Group 2	314909.1	79353.0		286001.4	77884.5	
MPV	Group 1	5.9	0.8	0.045	6.7	1.3	0.256
	Group 2	6.9	1.7		6.9	1.5	
NLR	Group 1	3.3	2.5	0.375	4.4	3.9	0.002
	Group 2	4.3	3.5		5.1	3.5	
PLR	Group 1	141.4	54.5	0.468	165.3	111.9	0.601
	Group 2	133.9	41.5		157.1	75.3	
RDW	Group 1	12.6	1.6	0.804	12.4	2.0	0.129
	Group 2	12.7	1.4		12.5	1.5	
hs-CRP	Group 1	0.3	0.5	0.011	2.0	4.4	0.382
	Group 2	2.2	4.2		2.2	4.3	
CPK-MB	Group 1	33.4	17.3	0.194	52.8	79.3	<0.0001
	Group 2	102.1	154.1		111.8	138.6	
Troponin I	Group 1	3.4	8.3	0.032	6.5	14.8	<0.0001
	Group 2	14.7	20.6		13.8	18.6	
ESR	Group 1	18.1	6.2	0.404	22.7	8.4	0.206
	Group 2	19.6	7.2		24.1	8.6	

CAD: coronary artery disease, group 1: population without CAD, group 2; population with CAD, WBC: white blood cell, MPV: mean platelet volume, NLR: neutrophil-to-lymphocyte ratio, PLR: platelet-to-lymphocyte ratio, PWBC: platelet white blood cell ratio, RDW: red cell distribution width, hs-CRP: high sensitivity C-reactive protein, CPK-MB: creatinine phosphokinase-MB, and ESR: erythrocyte sedimentation rate.

**Table 4:** Multivariate logistic regression analysis for coronary artery disease presence by various biochemical markers.

Variables	exp⁡(*B*)	Significance	95% CI for exp⁡(*B*)	
			Lower	Upper
WBC	1.000	0.030	1.000	1.000
Neutrophil	1.044	0.436	0.936	1.165
Monocyte	1.101	0.133	0.971	1.249
NLR	1.495	0.048	0.942	2.371
RDW	1.116	0.186	0.948	1.314
hs-CRP	1.031	0.399	0.960	1.109
CPK-MB	0.993	0.034	0.986	0.999
Troponin I	0.994	0.669	0.966	1.022
Constant	0.001	0.219		

exp⁡(*B*): exponentiation of the coefficients/odds ratios of the predictors, CI: confidence interval, WBC: white blood cell, NLR: neutrophil-to-lymphocyte ratio, RDW: red cell distribution width, hs-CRP: high sensitivity C-reactive protein, CPK-MB: creatinine phosphokinase-MB, and ESR: erythrocyte sedimentation rate.

**Table 5:** Receiver operative curve analysis of biochemical markers for CAD diagnosis.

Test result variable(s)	Area	Std. Error^a^	Asymptotic Sig.^b^	Asymptotic Sig.^b^	
				Lower bound	Upper bound
WBC	0.809	0.062	0.000	0.687	0.931
Neutrophil	0.821	0.056	0.000	0.714	0.932
Monocyte	0.697	0.064	0.022	0.572	0.823
NLR	0.823	0.056	0.000	0.712	0.931
RDW	0.713	0.064	0.014	0.587	0.839
hs-CRP	0.648	0.072	0.086	0.506	0.790
Troponin I	0.820	0.035	0.000	0.816	0.951

CAD: coronary artery disease, WBC: white blood cell, NLR: neutrophil-to-lymphocyte ratio, RDW: red cell distribution width, hs-CRP: high sensitivity C-reactive protein, CPK-MB: creatinine phosphokinase-MB, and ESR: erythrocyte sedimentation rate.  ^a^Under the nonparametric assumption.  ^b^Null hypothesis: true area = 0.5.

**Table 6:** Receiver operative curve analysis of NLR cut-off (2.13) for CAD diagnosis.

Statistic	Value	95% CI
Sensitivity	83.64%	78.67% to 87.86%
Specificity	63.46%	55.39% to 71.02%
Positive predictive value	79.79%	76.13% to 83.01%
Negative predictive value	69.23%	62.61% to 75.14%

CI: confidence interval.

**Table 7:** Association of acute coronary syndrome biomarkers with NLR.

Quartiles	hs-CRP	Mean NLR in hs-CRP quartile	CPK-MB	Mean NLR in CPK-MB quartile	Troponin I	Mean NLR in troponin I quartile
1st quartile	0.175	3.58	24	2.9	0.007	2.62
2nd quartile	0.45	4.88	40	4.07	0.974	4.31
3rd quartile	2.1	6.06	115	5.8	19.75	6.87

NLR, neutrophil-to-lymphocyte ratio; hs-CRP, high sensitivity C-reactive protein; CPK-MB, creatine phosphokinase-MB.
